# Influencing Factors of Survival in Hypopharyngeal Squamous Cell Cancer

**DOI:** 10.3390/jcm13133853

**Published:** 2024-06-30

**Authors:** Gábor Dénes Répássy, Dóra Hargas, András Molnár, Stefani Maihoub, László Tamás

**Affiliations:** 1 Department of Otorhinolaryngology and Head and Neck Surgery, Semmelweis University, Szigony u. 36., H-1083 Budapest, Hungary; g.repassy@gmail.com (G.D.R.); hargasdora@gmail.com (D.H.); stefaniem-9@hotmail.com (S.M.); tamas.laszlo@semmelweis.hu (L.T.); 2 Department of Voice, Speech and Swallowing Therapy, Semmelweis University; Vas u. 17., H-1088 Budapest, Hungary

**Keywords:** hypopharyngeal squamous cell cancer, survival, surgery, chemotherapy, radiotherapy, p16 expression, comorbidities

## Abstract

**Objectives:** This study examined the effects of various factors on survival in hypopharyngeal cancer, involving a total of 100 patients. **Methods:** Comorbidities, treatment modalities, survival times, and potential factors affecting survival were retrospectively analysed. The expression of p16 was also examined. A statistical analysis was conducted using IBM SPSS V25 software. **Results:** The mean overall survival time was determined to be 30.8 months. Smoking was observed in 95%, and regular alcohol consumption was reported in 75% of the cases. The expression of p16 did not significantly affect survival (*p* = 0.74) or the maximum tumour size (*p* = 0.21). The Kaplan–Meier method demonstrated significantly longer survival times (*p* = 0.047 *) in the group that underwent partial pharyngolaryngectomy with or without adjuvant therapy (median: 75.25 months, 95% CI: 31.57–118.93), compared to the other four treatment groups (i.e., total laryngectomy with pharyngectomy with or without adjuvant therapy, chemoradiation, chemotherapy, and radiotherapy). **Conclusions:** The study found that factors such as sex, comorbidities (e.g., type 2 diabetes and chronic obstructive pulmonary disease), TNM and stage, weight loss, smoking, and alcohol consumption did not have a significant effect on survival. In conclusion, the longest survival was observed after partial pharyngolaryngectomy with or without adjuvant therapy. Risk factors and comorbidities did not show a significant effect on survival. p16 expression was not a factor that affected either survival or tumour size.

## 1. Introduction

Hypopharyngeal cancers (HPCs) are not very common compared to other head and neck cancers [1], but they are difficult to manage due to their poor prognosis. HPCs are often diagnosed at an advanced stage, with around 60–80% of patients having lymph node metastases at the time of diagnosis [2]. Smoking and regular alcohol consumption are common in people with HPCs, and these are significant risk factors for developing this type of cancer [3,4], impacting the patient’s overall health [5]. Considering human papillomavirus (HPV) infection, some previous studies have indicated improved overall survival in HPV-positive HPCs; however, other research has not found a significant impact of HPV-positivity on survival [6]. The presence of HPV is indicated by the positive staining of the p16 protein. The p16 protein is a type of tumour suppressors and is an INK4 cell inhibitor. Normally, the retinoblastoma (Rb) protein binds to the E2F transcription factor, forming the Rb–E2F complex, which restricts cell proliferation. The p16 protein interacts with cyclin-dependent kinases 4 and 6, helping to maintain the Rb–E2F complex. However, in head and neck cancers, the loss of p16 function is characterised by genetic and non-genetic factors, particularly hypermethylation and loss of homozygosity. The HPV virus demonstrates its oncogenic properties by affecting the cell cycle, including an impact on the Rb and p107 and -130 molecules. While there is a connection between overexpression of p16 and HPV-positivity, it is essential to note that the two are not always the same. In some cases of p16-positive head and neck cancers, HPV-negativity may still be observed [7,8,9]. A better prognosis is observed for p16-positive head and neck cancers, according to previous studies. Hence, the presence of p16 expression may impact an individual’s survival [10].

Research has shown that the overall 5-year survival rate for HPCs ranges between 25 and 50%, depending on the disease stage and treatment methods used, with the best survival seen in cases where surgery is performed [11].

In terms of surgical treatment for HPCs, it is important to consider some fundamental facts. Based on the classification of surgeries for HPCs, there are internal and external approaches (including partial pharyngolaryngectomies and total laryngectomy with pharyngectomy), with or without flap reconstructions. For early-stage HPCs, it remains uncertain whether extensive surgery is necessary. Therefore, the necessity of partial pharyngectomy remains unclear, and radiotherapy is considered equally effective in the treatment of early-stage HPCs in terms of curability and potential complications [12].

When considering external approaches to partial surgeries, partial pharyngectomy with or without partial laryngectomy can be used for T1–T2 and some T3 HPCs. For T1 and some T2 lateral pharyngeal wall cancers, it is possible to remove the tumour using an external approach that does not involve laryngeal surgery. Some piriform sinus cancers may be small enough to be treated with a lateral partial pharyngectomy, with the option for primary or flap reconstructions [13]. Infrahyoid pharyngotomy is typically performed in such cases. However, for locally advanced cancers, hyoid bone resection and suprahyoid pharyngotomy may be necessary. However, patient selection must be carried out with great care [14]. Since the 1960s, supracricoid hemipharyngo-laryngectomy has been used for treating cancers, specifically in the piriform sinus, when the vocal cord is cancer-free and mobile. This surgical procedure involves removing the lateral parts of the thyroid cartilage, the arytenoid cartilage on the affected side, and the posterior part of the vocal cord. A portion of the lateral wall of the piriform sinus is retained for closure [15,16]. To treat tumors infiltrating the medial wall of the piriform sinus and extending into the supraglottic and marginal regions, the procedure of supraglottic hemipharyngo-laryngectomy may be performed. The aforementioned surgical procedures have been shown to be good alternatives to total laryngectomy, with similar survival rates. Nevertheless, the quality of life is considerably better following hemipharyngo-laryngectomy in comparison to total laryngectomy. The latter involves the complete surgical removal of the larynx and the resultant disruption of the airways. Subsequently, respiration becomes feasible through a tracheostomy tube [17]. Although transoral approaches to cancers are promising and beneficial, external partial surgeries are still essential. They provide an opportunity for functional preservation, even in advanced cases of HPC [18].

When considering partial surgeries, an alternative approach using an endoscope can be applied. Endoscopic excision using carbon dioxide lasers allows for better functional results. However, patient selection is more limited. Internal tumour incision (transoral partial hypopharyngectomy) can mainly be used for locally non-advanced T1 and some T2 tumours, as this type of exploration (using a supraglottiscope in intubated patients) is limited and cannot be applied in locally advanced tumours. This procedure can be performed using a carbon dioxide laser, transoral robotic surgery, or ‘cold steel’. Although laser-assisted techniques can be advantageous and promising [19], they can also result in postoperative complications, such as local infections, emphysema, cutaneous fistulae, postoperative bleeding, dyspnoea, or swallowing difficulties. Considering postoperative functionality and rehabilitation, in these cases, tracheostomy or feeding through a nasogastric tube is unnecessary. In cases of locally advanced HPC, it is advisable to consider referring patients for chemoradiation. If there are recurrences or persistent tumours, a salvage surgery such as pharyngolaryngectomy or total laryngectomy with pharyngectomy and neck dissection may be necessary to address the complications and loss of functionality [20].

Since the late 1990s, studies have compared surgical and non-surgical treatment options for HPCs. Initially, no significant differences were found between surgical options and chemoradiation therapies [21,22]. Other studies have also not shown differences in overall and disease-specific survival (DSS) when comparing surgery, postoperative radiotherapy, salvage surgery, and radiotherapy in T2–T4a HPCs [23,24]. However, some studies have identified radical surgery as the best treatment option for locally advanced HPCs. Consequently, further research is needed to analyse the effects of different treatment options on HPC survival [25]. Therefore, our study aims to analyse the difference in survival between surgical and non-surgical treatments for HPC, including potential factors that may impact outcomes. Specifically, we are investigating the surgical treatment option of partial pharyngolaryngectomy, which has received less attention, as well as the expression of p16 in HPCs, which is not yet well understood.

## 2. Materials and Methods

### 2.1. Study Population and Design

In this retrospective investigation, a total of 100 participants (87 men and 13 women, mean age ± SD, 68.05 ± 5.63 years) with HPCs were included. The medical records of each participant were thoroughly reviewed in the electronic medical record system of Semmelweis University, including basic demographic data, diagnosis, histological examinations, TNM (i.e., tumour, regional nodal metastasis, and distant metastasis) classification and staging (Table 1), and any comorbidities. All participants were diagnosed and treated according to the latest National Comprehensive Cancer Network^®^ (NCCN) Guidelines for Head and Neck Cancers [26]. The investigations and therapies were carried out at the Department of Otorhinolaryngology and Head and Neck Surgery of Semmelweis University. Participants with incomplete data were excluded, and all participants were followed up for at least one year. The types of treatment for HPCs were categorised into five categories, i.e., total laryngectomy with pharyngectomy with or without adjuvant therapy, partial pharyngolaryngectomy with or without adjuvant therapy, chemoradiation, chemotherapy, and radiotherapy.

Considering the full oncological evaluations, all patients underwent laryngeal endoscopy and laryngomicroscopy under general anaesthesia to conduct a biopsy and identify the local extent of the tumour. Staging involved CT examinations of the neck, chest, abdomen, and pelvis with contrast, or PET-CT examinations and CT examinations of the neck using contrast. The histological type of the tumours was categorised as squamous cell carcinoma or basaloid squamous cell carcinoma. Additionally, the p16 expression of the tumour cells was considered. Staging classification for hypopharyngeal cancers was determined using the latest American Joint Committee on Cancer (AJCC) staging manual [27]. The maximum tumour size was determined as the longest diameter of the tumour mass in millimetres, based on the surgical specimen or medical imaging in the non-surgery treatment groups. Regarding the general characteristics of this population, smoking habits, regular alcohol consumption, comorbidities—e.g., type 2 diabetes mellitus (T2DM) and chronic obstructive pulmonary disease (COPD)—and weight loss were all considered. The time between the first symptoms and diagnosis, and the OS from the tumour diagnosis were calculated in months. The study was conducted according to the guidelines of the Declaration of Helsinki and approved by the Semmelweis University Regional and Institutional Committee for Science and Research Ethics: SE IKEB 105/2014.

### 2.2. p16 Immunohistochemistry

To conduct p16 immunohistochemistry, 4 µm histology slides were stained using a Benchmark Ultra Plus automated system (Roche, Basel, Switzerland). The first step involved deparaffinising the histology slides in EZ Prep Solution (Roche, Basel, Switzerland). Heat-induced epitope retrieval was carried out using a cell conditioning solution with a pH of 9 (Roche, Basel, Switzerland) at 95 °C for 30 min. To inhibit endogenous peroxidase activity, one drop of UV INHIBITOR (Roche, Basel, Switzerland) was applied and left to incubate at 37 °C for 6 min. A primary monoclonal antibody against p16 INK4 (Cell Marque, Rocklin, CA, USA) was then applied at 37 °C for 1 h with a dilution ratio of 1:100. Visualisation of the bound primary antibodies was achieved using the OptiView Amplification Kit (Roche, Basel, Switzerland). Haematoxylin II (Roche, Basel, Switzerland) was used for nuclear counterstaining. All washing steps were performed using diluted Reaction Buffer Concentrate (Roche, Basel, Switzerland). Positive p16 staining was identified if a distinct cytoplasmic and nucleolar positive reaction was observed in at least 70% of the tumour tissue [28].

### 2.3. Treatment Options

The best treatment strategy was determined for each patient based on the latest NCCN guidelines, considering individual patient characteristics. For early, non-advanced tumours (T1–2 and N0), radiation therapy or surgery is recommended. Based on the postoperative histology results, surgery may be performed without the need for postoperative radiation in some cases, particularly with R0 resections. In T1 and T2 cases, the best approach when lymph node positivity (N+) is observed is either surgery or chemoradiation. In cases of T3 and T4 without lymph node involvement, either surgery or chemoradiation should be administered. Surgery and postoperative chemoradiation should be offered for any *T* stages when N2 or N3 disease is present.

In terms of surgical options, total laryngectomy and partial pharyngectomy are the best choices for locally advanced tumours (i.e., T3 or T4), with adjuvant chemoradiation in cases of significant lymph node involvement. Surgery is necessary for T4 tumours, while T2 HPCs may require partial pharyngolaryngectomy. Chemoradiation is recommended for cases involving metastases, R1 resections, lymphovascular invasion, or extracapsular spreading.

Adjuvant treatment is necessary for N2 and N3 cases, R1 resection, and extra-nodal or perineural spreading. Adjuvant radiation is recommended for T3 and T4 cases, while adjuvant chemotherapy is not used independently. Chemotherapy is only considered as monotherapy in cases where patients do not consent to radiation therapy. In terms of radiotherapy, a total radiation dose of 66–70 Gy is typically applied in 33–35 fractions over six to seven weeks (2 Gy/fractions). Postoperatively, radiation is used in locally advanced tumour cases with a dosage of 54 to 60 Gy for complete surgical resection and 60 to 66 Gy for R1 resection. A dosage of 50 to 66 Gy is used for the lymph node areas based on extracapsular spreading. It is essential to begin radiotherapy promptly, ideally within two months after the surgical procedure. Cisplatin is used in body-surface-area-based dosing for chemotherapy, specifically 100 mg/m^2^ intravenously every three weeks. Adjuvant chemoradiation is recommended in high-risk cases where there is histological evidence of regional lymph node metastases, extracapsular extension of the nodal disease, or microscopically involved tumour margins. Chemotherapy toleration is assessed using the ECOG (Eastern Cooperative Oncology Group) scale, and routine laboratory testing, including white blood cell and platelet counts and creatinine clearance, is performed before chemotherapy [29,30].

### 2.4. Statistical Analysis

All statistical analyses were conducted using IBM SPSS V25 software (IBM Corporation, Armonk, NY, USA). Data normality was assessed using the Shapiro–Wilk test. Continuous variables were reported as either the mean ± SD or median, depending on the distribution of the data. The Mann–Whitney *U* and Kruskal–Wallis tests were used to identify significant differences between the groups. Survivorship curves (Kaplan–Meier curves) were plotted, and the log-rank (Mantel–Cox) test was employed to assess the impact of different factors on survival. The Chi-square test was utilised for categorical analysis. Additionally, multinomial logistic regression was carried out. The Spearman’s correlation test was used to examine the relationships between the parameters. The significance level was set at *p* < 0.05.

## 3. Results

Table 1 summarises the basic information of the study population.

In the current study, Table 1 shows that there was a higher number of male patients (87%) compared to female patients, which is commonly seen in head and neck cancers. The mean age of the patients was approximately 68 years, indicating that HPC is usually diagnosed in older individuals. The mean OS time from tumour diagnosis was 30.8 months, with the longest-surviving patient living for 216 months (18 years) and the shortest-surviving patient living for ten months. Smoking and regular alcohol consumption were observed in 95% and 70% of the participants, respectively, making them the most significant risk factors for HPCs. No significant differences were found between the treatment groups concerning patients’ age or sex distribution, the time between the first symptoms and diagnosis, weight loss, smoking, and alcohol consumption. However, there were statistically significant differences in the *T* stage (*p* = 0.00004 *) and the *M* stage (*p* = 0.0009 *) when the TNM categories were compared using the Chi-square test. No significant difference was found when the *N* stages were compared.

In the next step of the investigation, the study analysed the difference in survival considering different factors. First, the survival and tumour size differences in the p16-positive and -negative groups were examined.

**Figure 1:**
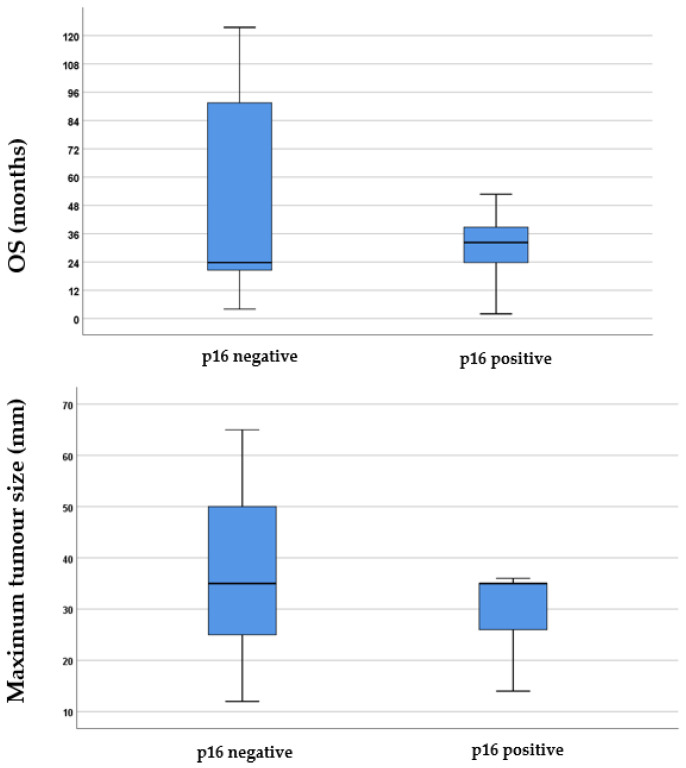
Boxplots illustrating the difference in p16 expression regarding both survival and the maximum tumour size in millimetres. In the boxplots, the boxes represent the middle 50% of the data, while the whiskers represent the upper and lower 25%. The median values are indicated by the black lines that divide the boxes into two parts. The differences were analysed using the Mann–Whitney *U* test. OS = overall survival.

The study found that 11% of the total population tested positive for p16 expression. Specifically, 4 out of 29 (13.79%) of those who underwent total laryngectomy with or without adjuvant therapy, 1 out of 14 (7.14%) who had partial pharyngolaryngectomy with or without adjuvant therapy, 4 out of 10 (40%) receiving chemoradiation, none out of 3 (0%) who received chemotherapy, and 2 out of 14 (14.28%) treated with radiation showed p16 expression positivity. Figure 1 shows that although patients with p16-negative tumours tended to have longer survival times, the statistical analysis using the Mann–Whitney *U* test (*p* = 0.74, *Z*-score: −0.34) did not find a significant difference in survival times between the two groups. Additionally, although there were slightly larger tumour sizes in p16-negative cases, this difference was not statistically significant according to the Mann–Whitney *U* test (*p* = 0.21, *Z*-score: 1.23).

**Figure 2:**
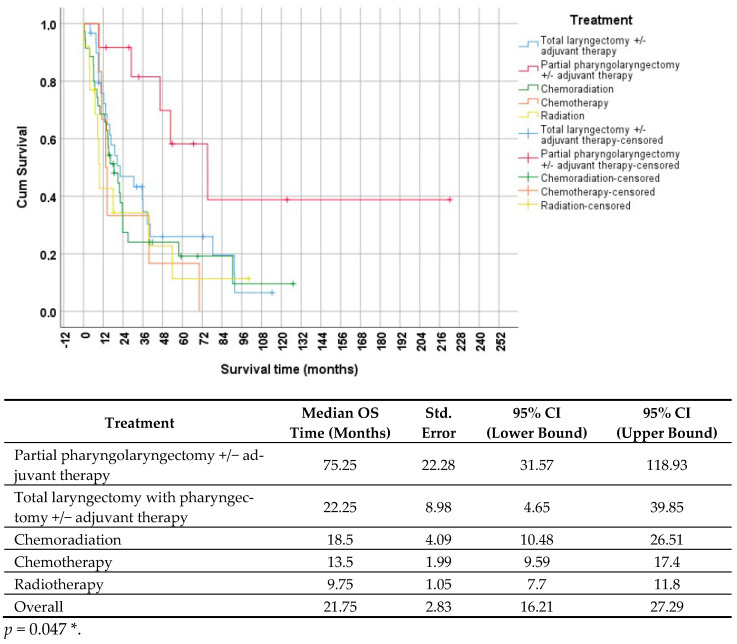
Survivorship (Kaplan–Meier) curves, indicating survival time based on different therapies. The curve is divided into five groups; the red line represents partial pharyngolaryngectomy with or without adjuvant therapy, the blue line represents total laryngectomy with pharyngectomy with or without adjuvant therapy, the yellow line represents radiotherapy, the orange line represents chemotherapy, and the green line represents chemoradiation. The table below the survivorship curve displays the results of the statistical analysis. The *p*-value was calculated using the log-rank (Mantel–Cox) test (*p* < 0.05 *). CI = confidence interval; OS = overall survival; Std. = standard. The asterisk (*) indicates a statistically significant difference.

Figure 2 illustrates the survivorship curves for various treatment modalities.

In Figure 2, it is evident that patients who underwent partial pharyngolaryngectomy with or without adjuvant therapy had the longest median survival time of 75.25 months (95% CI: 31.57–118.93). Conversely, those who received radiation monotherapy had the shortest survival time of 9.75 months (95% CI: 7.7–11.8). The statistical analysis, using the log-rank test, indicated a significant difference between the groups (*p* = 0.047 *), implying a notably longer survival time for patients who underwent partial pharyngolaryngectomy.

**Figure 3:**
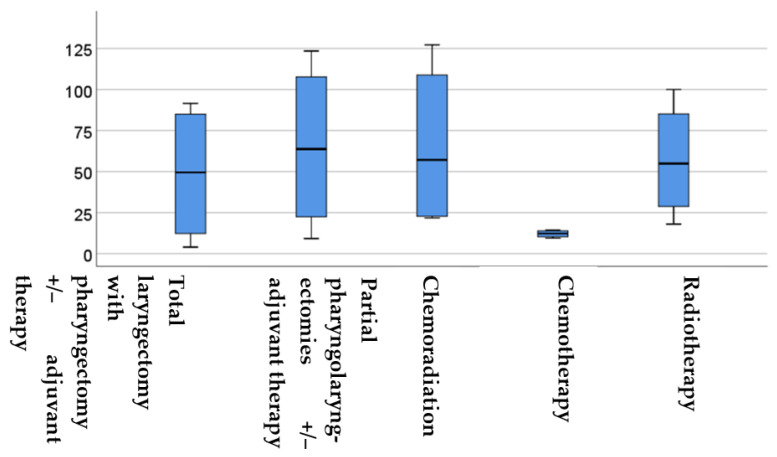
Boxplot displaying the survival times in months for each group. The boxes show the middle 50% of the data and the whiskers depict the upper and lower 25%. The black lines within the boxes represent the median values. The differences were analysed using the Kruskal–Wallis test. OS = overall survival.

In Figure 3, the survival times of the five therapy groups are compared.

Figure 3 demonstrates that chemotherapy led to significantly lower survival times compared to the other four therapy groups. The statistical analysis using the Kruskal–Wallis test showed a statistically significant difference between the groups (*p* = 0.004 *, *H* test statistic = 15.12). Further analysis using the Mann–Whitney *U* test revealed significant differences between the various therapy groups. Specifically, there was a significant difference in survival between the partial pharyngolaryngectomy with or without adjuvant therapy and total laryngectomy with or without adjuvant therapy (*p* = 0.04 *, *Z*-score: −2.02), partial pharyngolaryngectomy with or without adjuvant therapy and chemoradiation (*p* = 0.0016 *, *Z*-score: −3.15), partial pharyngolaryngectomy with or without adjuvant therapy and chemotherapy (*p* = 0.006 *, *Z*-score: 2.7), as well as radiotherapy (*p* = 0.007 *, *Z*-score: 2.69) groups. These results indicate significantly longer survival in the case of partial pharyngolaryngectomy with or without adjuvant therapy.

In addition to comparing the therapies, the prediction of other factors on survival was also analysed, and the results are presented in Table 2.

The results in Table 2 indicate that none of the examined factors significantly predicted survival in HPC. There were no significant differences in survival times between men and women, and similar results were observed when considering smoking habits and regular alcohol consumption. When Spearman’s test was used to analyse the correlation between pack-years and overall survival, no significant correlation was found (rho = 0.097, *p* = 0.491). Additionally, comorbidities such as COPD and T2DM were not found to be predictive factors for survival. Surprisingly, the tumour stage, as determined by TNM and grading, was not found to significantly predict survival times.

## 4. Discussion

In this study, we analysed the most important factors affecting survival in HPC. We found that patients who underwent partial pharyngolaryngectomy with or without adjuvant therapy had significantly longer OS compared to those who chose other treatment options. Additionally, we observed no significant difference in terms of survival and tumour size in the p16-positive and -negative groups. General risk factors such as alcohol consumption and smoking, and certain comorbidities like T2DM, COPD, and weight loss, did not significantly predict survival in HPC.

One of the most common cancers worldwide is head and neck cancer, affecting nearly a million people each year [31]. Squamous cell carcinomas of the head and neck can involve the lips and oral cavity, the oropharynx and nasopharynx, and the larynx and hypopharynx. This article primarily focuses on squamous cell carcinomas that arise in the hypopharynx, and the potential factors influencing their survival. The highest rates of squamous cell carcinoma globally are found in Central and Eastern Europe (i.e., 3.9 per 100,000) [32]. Our study population showed a male predominance, which is consistent with other recent studies [33] and is generally observed in head and neck malignancies. The average age of the patients was approximately 68 years, in line with similar studies but slightly higher [34,35,36]. The population’s exposure to social and behavioral risk factors, particularly alcohol consumption and smoking, has played a crucial role in the development of HPC and has contributed to a decrease in OS rates, as indicated by previous findings [37]. Our study also reported a high frequency of abuse, which supports this statement. Based on the statistical analysis, their survival prediction was determined to be not significant. Although the coincidence of some comorbidities, such as T2DM, weight loss, and COPD, is high in HPC, in the current investigation, they did not significantly predict the survival rates.

The influence of the immunohistochemical factor p16 on HPC was analysed for its potential value in predicting prognosis and survival. Our study population exhibited an 11% positivity for p16 expression, a relatively low ratio but consistent with previous study findings [38,39]. Furthermore, this study found no significant difference in survival between the p16-positive and -negative groups. This lack of a significant difference was attributed to the fact that the p16 protein did not exhibit widespread cytoplasmic and nuclear presentation in the hypopharyngeal tumour cells and, therefore, did not have significant oncogenic effects in HPC. Consequently, there is no significant correlation with high-risk HPV infections in HPC [40,41]. Initially, it was assumed that p16-negative tumours might lead to longer survival times and slightly larger tumour sizes, but no statistically significant differences were found. Other studies have shown conflicting results, with some indicating a higher survival rate for p16-positive patients [42] and others showing no correlation with survival [43]. It is noted that more extensive studies adjusting for prognostic factors are needed in future research to further understand the role of p16 in HPC.

In our study, we explored a wide range of treatment options for HPC, including various surgical techniques (such as total or partial pharyngolaryngectomies with or without adjuvant therapy), chemotherapy, and radiation. The median survival for our study population was 21.75 months. Notably, the longest survival was observed in the group that underwent partial pharyngolaryngectomy with or without adjuvant therapy, while the shortest survival was seen in the radiation therapy group. Our survivorship curve analysis revealed significantly longer survival in the partial pharyngolaryngectomy group compared to other therapy groups. Our findings are consistent with previous research, which also demonstrated that patients who received surgical therapy alone had a better survival outcome compared to those who received radiation without surgery [12]. Additionally, Galeano Machuca et al. found better 5-year OS in advanced HPC patients who underwent surgery as the first-line treatment [44]. While organ preservation strategies are feasible in advanced HPCs, the results for squamous cell carcinoma are less promising. Attempts to preserve the larynx in these cases have led to higher rates of local and regional recurrence [45]. In their study, Takes et al. compared the OS of patients undergoing surgeries with different techniques. They found that the 3-year survival rates were 59% for the partial-pharyngolaryngectomy-treated group, 36% for the total-laryngectomy-treated group, and 11% for the palliation group [46]. Partial pharyngolaryngectomy is a minimally invasive surgical procedure developed to reduce trauma and preserve laryngeal function in certain cases of HPC. These surgeries provide a minimal invasive option compared to total laryngectomy, enabling patients to maintain vital functions like speech, breathing, and swallowing, ultimately enhancing their quality of life. Layton et al. compared the functional outcomes after total laryngectomy and pharyngolaryngectomy. They concluded that partial pharyngectomy seems to offer some advantages over total laryngectomy in terms of functional outcomes. The study suggests that patients undergoing partial pharyngolaryngectomy tend to have better speech and swallowing outcomes than those undergoing more extensive resections, such as total laryngectomy [47]. Specifically, the results indicate that patients with less radical resections have a higher normal speech rate and better swallowing outcomes. Cortese et al. pointed out that partial pharyngolaryngectomy can lead to positive oncological outcomes. In their study, 91% of patients experienced tracheostomy closure, and 75% were able to resume oral feeding [48].

The results of the present investigation emphasise some crucial clinical facts that should be considered when treating patients with HPC. Firstly, due to the possibility of HPC recurrence, surgical treatment options should be the primary choice, considering technical reasons such as soft tissue fibrosis after radiotherapy, particularly in cases of salvage surgeries [49], making surgeries more complicated. Additionally, chemoradiation prior to surgery affects the available surgical options. After chemoradiation, only total laryngectomy is a viable surgical treatment, while partial pharyngolaryngectomies cannot be performed. The preservation of organs and functions is crucial, and this can be achieved through partial pharyngolaryngectomies. However, there are possible complications with chemoradiation, such as radiation-induced fibrosis, which can affect organ function. In such cases, the use of partial pharyngolaryngectomy may be advantageous.

This study had some limitations. Firstly, it was a retrospective investigation, but this allowed for long-term patient follow-up. Secondly, the treatment options were categorised into groups, which did not allow for a detailed analysis of the therapies. Furthermore, due to the retrospective design of this investigation, the potential influence of other factors could not be conclusively ruled out. However, because a relatively small number of patients received chemotherapy as a monotherapy, there was an uneven distribution between the treatment groups, which could have introduced bias. Future studies should consider including more cases of HPC. Additionally, analysing the patients’ quality of life after treatment could provide a more comprehensive perspective, using assessment scales for quality of life, language function, and swallowing function. Furthermore, dividing the treatment options into more specific groups could improve the usability of the results.

## 5. Conclusions

When considering treatment options, this research found that the longest survival was observed after partial pharyngolaryngectomies. These procedures are crucial for preserving organ function. Therefore, when local tumour spreading allows, partial pharyngolaryngectomies should be considered, considering the impact of chemoradiation on organ functioning and the available surgical therapy options. It was also found that risk factors and comorbidities were not significant predictors of survival. Additionally, there was no difference in survival and tumor size between the p16-positive and -negative groups. Given the study design, future research should investigate the factors influencing HPC survival.

## Figures and Tables

**Table 1:** Participants’ basic characteristics. Continuous variables are presented as mean ± SD, while whole numbers (*n*) and percentage (%) values are also provided. The *p*-values indicate statistically significant differences between four treatment groups. OS = overall survival; SD = standard deviation. ^a^ Kruskal–Wallis test, ^b^ Chi-square test (*p* < 0.05 *). The asterisk (*) indicates a statistically significant difference.

Category	Overall	Total Laryngectomy with Pharyngectomy +/− Adjuvant Therapy (*n* = 29)	Partial Pharyngolaryngectomy +/− Adjuvant Therapy (*n* = 14)	Chemoradiation (*n* = 40)	Chemotherapy (*n* = 3)	Radiotherapy (*n* = 14)	*p*-Value
Age (years, mean ± SD)	68.05 ± 5.6	70.81 ± 4.08	69.4 ± 3.64	64.7 ± 6.83	71 ± 4	69.2 ± 6.5	0.07 ^a^
Sex distribution (men/women)	87/13	25/4	12/2	35/5	3/0	12/2	0.97 ^b^
Time between first symptoms and diagnosis (months, mean ± SD)	5.4 ± 4	6.5 ± 6.9	5.59 ± 3.69	4.9 ± 4.3	5 ± 1.75	5.04 ± 2.46	0.64 ^a^
Weight loss (kg, mean ± SD)	4 ± 4.2	5.14 ± 3.06	3.75 ± 3.5	3.23 ± 3.41	3.33 ± 1.1	7.16 ± 7.5	0.1 ^a^
Smoking, *n* (%)	95 (95%)	29 (100%)	12 (85.7%)	33 (82.5%)	3 (100%)	14 (100%)	0.5 ^b^
Regular alcohol consumption, *n* (%)	70 (70%)	29 (100%)	14 (100%)	31 (77.5%)	0	13 (92.8%)	0.0022 ^b,^*
‘*T*’ stage, *n* (%)							0.00004 ^b,^*
1	11	0 (0%)	2 (14.3%)	3 (7.5%)	1 (33.3%)	5 (35.7%)	
2	22	3 (10.3%)	10 (71.4%)	7 (17.5%)	1 (33.3%)	1 (7.14%)	
3	29	11 (37.9%)	1 (7.14%)	10 (25%)	1 (33.3%)	6 (42.9%)	
4	38	15 (51.8%)	1 (7.14%)	20 (50%)	0 (0%)	2 (14.26%)	
‘*N*’ stage, *n* (%)							0.77 ^b^
0	20	2 (6.9%)	5 (35.7%)	9 (22.5%)	0 (0%)	4 (28.5%)	
1	14	4 (13.8%)	2 (14.3%)	6 (15%)	1 (33.3%)	1 (7.1%)	
2	45	17 (58.6%)	6 (42.8%)	15 (37.5%)	1 (33.3%)	6 (42.9%)	
3	21	6 (20.7%)	1 (7.14%)	10 (25%)	1 (33.3%)	3 (21.5%)	
‘*M*’ stage, *n* (%)							0.0009 ^b,^*
0	95	29 (100%)	14 (100%	39 (97.5%)	1 (33.3)	12 (85.7%)	
1	5	0 (0%)	0 (0%)	1 (2.5%)	2 (66.7%)	2 (14.3%)	

**Table 2:** Multinomial logistic regression analysis of the potential factors predicting survival. CI = confidence interval; COPD = chronic obstructive pulmonary disease; Std. = standard; T2DM = type 2 diabetes mellitus; *T* = tumour; *N* = regional nodal metastasis; *M* = distant metastasis. Exp. (*β*) shows the odds ratio. The significance level was set at *p* < 0.05.

Factor	*β*	Std. Error	*p*-Value	Exp. (*β*)	95% CI (Lower Bound)	95% CI (Upper Bound)
Smoking	−16.87	1.95	0.998	0.890	0.030	26.597
Regular alcohol consumption	0.98	0.839	0.239	2.686	0.518	13.917
COPD	−1.098	1.025	0.284	0.333	0.045	2.484
T2DM	17.754	0.000	0.219	3.75	0.455	30.88
p16 expression	−1.409	2.000	0.481	0.244	0.005	12.308
Weight loss	0.061	0.759	0.936	1.063	0.240	4.703
Sex	−1.394	1.350	0.302	0.248	0.018	3.496
Grade 1	0.197	0.901	0.827	1.217	0.208	7.114
Grade 2	0.363	0.552	0.511	0.696	0.236	2.053
Grade 3	0.296	0.527	0.574	1.345	0.478	3.780
*‘T’*	−0.283	0.194	0.147	0.755	0.516	1.104
*‘N’*	0.111	0.140	0.429	1.117	0.849	1.469
*‘M’*	0.470	1.147	0.682	1.6000	0.169	15.163

## Data Availability

The data presented in this study are available from the corresponding author upon reasonable request.
